# Searching for alpha-1 antitrypsin deficiency in patients with bronchiectasis: reducing idiopathic cases

**DOI:** 10.36416/1806-3756/e20250449

**Published:** 2025-11-19

**Authors:** Sara Qutubuddin, Rodrigo Cavallazzi

**Affiliations:** 1. Division of Pulmonary, Critical Care, and Sleep Disorders, Department of Medicine, University of Louisville, Louisville, KY, USA

**Figure 1:**
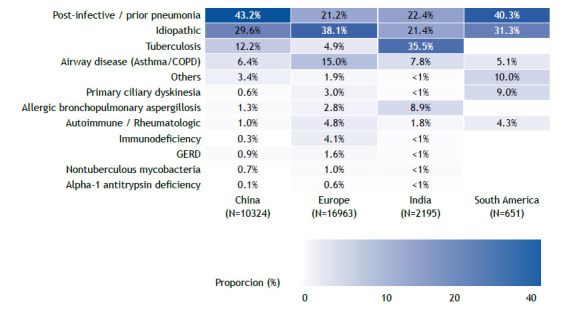
Heatmap graph showing the etiology of bronchiectasis in different cohorts across the globe.

There has been substantial progress in our understanding of bronchiectasis in recent years. It is well established that bronchiectasis is characterized by a neutrophilic airway inflammation, but a new finding is the recognition that 20% of patients with bronchiectasis have an eosinophilic profile, which has implications in the risk of exacerbations.^1^ Research on microbiome has illustrated the importance of microbial interactions and the potential role of commensal microbes in the pathogenesis of exacerbation.^2^ Multicenter cohorts have informed us about the most common causes of bronchiectasis in different areas of the globe (Figure 1).^3^ ^-^ ^6^ In South America, the most common causes of bronchiectasis in a study^4^ that included 651 patients were post-infective (40.3%), idiopathic (31.3%), primary ciliary dyskinesia (9%), airway disease (5.1%), rheumatologic disease (4.3%), and other causes (10%).In that cohort, the post-infective etiology included both tuberculosis and other lung infections.^4^

One of the etiologies of bronchiectasis is alpha-1-antitrypsin (A1AT) deficiency, an autosomal codominant disease characterized by a mutation leading to an amino acid change in the A1AT. This results in misfolding of the protein, rendering it unable to perform its usual function of inhibiting proteinases. Unchecked, the proteinases may lead to lung parenchymal damage and immune dysfunction. Furthermore, the polymerization of the enzyme in the hepatocytes can cause liver disease. In addition to emphysema and liver disease, patients are also at an increased risk of developing bronchiectasis and necrotizing panniculitis.^7^

In a registry cohort of 418 patients with the genotype PiZZ A1AT deficiency who underwent chest CT scanning, the results of which were abnormal in 82% of the patients.^8^ The most common abnormalities included emphysema alone in 45%, bronchiectasis plus emphysema in 27%, and bronchiectasis alone in 9% of the patients. Overall, bronchiectasis was present in 36% of the patients.^8^ In a clinic for patients with COPD in Brazil, 27 patients with A1AT compatible mutation were identified. Of these, bronchiectasis was present in 14 patients (52%).^9^ A corollary to these findings is the prevalence of A1AT deficiency in patients with bronchiectasis. This clinically relevant question is expected to be influenced by the prevalence of A1AT deficiency in the general population where patients are being seen.

In a multinational study,^10^ 2,620 dried blood or buccal swab samples from patients in Brazil with suspected A1AT deficiency underwent allele-specific genotyping for the 14 most common deficiency variants of the *SERPINA1* gene. Further testing with gene sequencing was conducted if no mutation was found, or if a mutation was found in the heterozygous status and the A1AT level was lower than 60 mg/dL, or per clinician request. An A1AT deficiency mutation was present in 28% of the samples. The most frequent genotypes were PiMS (12.7%), PiMZ (7.4%), PiSS (0.8%), PiSZ (1.6%), and PiZZ (3%). Allele percentages were S (15.2%), Z (12.6%), rare alleles (2.5%), null alleles (0.2%), and new alleles (0.2%). Bronchiectasis was the second most common reason for testing patients for A1AT. The prevalence of mutation in the Brazilian cohort was higher than that of other South American countries for which data were available (Argentina, Chile, and Colombia), but the non-random nature of data collection precludes an accurate comparison among countries. For example, in the Brazilian cohort, a substantially higher proportion of patients were tested due to a family history of A1AT deficiency in a biological relative.^10^

Another important study conducted in Brazil evaluated the prevalence of A1AT deficiency and the allele frequency in 926 patients with COPD from five states covering four Brazilian regions.^11^ All patients had A1AT levels measured in dried spot blood samples. For those with dried spot blood A1AT levels < 2.64 mg/dL, measurement of A1AT levels in the serum was conducted. For those with serum A1AT levels < 113 mg/dL, genotyping was performed. Genetic sequencing was conducted when serum A1AT levels were < 113 mg/dL, but no S and Z alleles were detected in genotyping. The prevalence of A1AT deficiency was 2.8%, whereas that of the PiZZ genotype was 0.8%. In patients with serum A1AT levels < 113 mg/dL, the most frequent allele was Z (53.8%). The authors of the study pointed out that the prevalence of A1AT deficiency in Brazil was similar to that of other countries.^11^ However, it is possible that the prevalence of A1AT deficiency in Brazilian patients with COPD is even higher than what was reported in that study, because genotyping was only triggered when serum A1AT levels were < 113 mg/dL.

In this issue of the *Jornal Brasileiro de Pneumologia*, Sokoloski et al.^12^ included 136 adult outpatients without cystic fibrosis who were seen in an academic referral center in Southern Brazil in their study. Patients underwent A1AT level measurement in serum and genotyping of the *SERPINA1* gene for 14 common variants from buccal swab samples. Serum A1AT levels were classified as normal (≥ 116 mg/dL), intermediate (57-115 mg/dL), and severely reduced (< 57 mg/dL). The authors of the study considered A1AT deficiency as a definitive etiology for bronchiectasis in the presence of genotypes that cause severe A1AT deficiency, such as PiZZ, PiSZ, and PiZM(Malton). Serum levels of A1AT were < 116 mg/dL in 28 patients (20.6%) of the cohort. The levels were < 57 mg/dL in 3 patients (2.2%) and between 57 and 115 mg/dL in 25 patients (18.4%). At least one *SERPINA1* mutation was detected in 35 patients (25.7%). Pathogenic A1AT variants were detected in 17.4% of patients with serum A1AT levels ≥ 116 mg/dL, 64% of whom with levels between 57 and 115 mg/dL, and in 100% of patients with levels < 57 mg/dL. The overall cohort had a high proportion of patients with emphysema (33%). However, the proportion of emphysema was not significantly different between patients with A1AT mutations and those without it (28.6% vs. 34.7%). Additionally, among the patients with A1AT mutations and emphysema, only 30% had the panlobular type of emphysema. A1AT deficiency, as per author’s definition, was the etiology of bronchiectasis in 2.9% of patients.^12^

There are important take-home messages from the study by Sokoloski et al.^12^ that apply to this particular cohort of patients with bronchiectasis: (1) normal A1AT levels did not exclude pathogenic variant alleles; (2) emphysema was not more prevalent in patients with A1AT mutations; (3) the typical panlobular pattern of emphysema was not always present in patients who had A1AT mutations and emphysema; (4) the systematic investigation for A1AT is the likely reason why the prevalence of A1AT deficiency was higher than that reported in the major international cohorts of patients with bronchiectasis^3^ ^-^ ^6^; and (5) the comprehensive genotyping allowed the authors to detect a rare genotype (Pi*MI) in a young patient with a normal A1AT level and bronchiectasis of undetermined etiology. The study has limitations that the authors recognized. For example, its single-center nature limits generalizability, particularly in a large and diverse country as Brazil. The relatively small sample size undermines the precision of the estimates. The proportion of patients with emphysema was higher than that in the major published cohorts of patients with bronchiectasis,^3^ ^-^ ^6^ highlighting the unique aspects of the practice where the study was conducted.

The study has important clinical implications. Currently, most guidelines or consensus on bronchiectasis recommend testing A1AT deficiency conditional on the presence of emphysema or basal emphysema or panlobular emphysema.^13^ ^-^ ^15^ The exception is the Brazilian consensus, which recommends routine investigation for A1AT deficiency in such patients (Chart 1).^16^ However, if the investigation for A1AT deficiency is predicated on the presence of emphysema, many patients (probably the majority) with A1AT mutations will be missed. A more reasonable approach would be to test patients with bronchiectasis for A1AT deficiency in the presence of emphysema or if the cause of bronchiectasis has yet to be clearly established. As shown in Figure 1, large multicenter cohorts of patients with bronchiectasis consistently show that an etiology for bronchiectasis cannot be established in approximately one third of patients. Another important implication is that reliance on isolated A1AT levels is likely to miss many patients with pathogenic A1AT variants, which illustrates the importance of adding genotyping to the investigation. Moving forward, it will be interesting to conduct a similar study in a multicenter Brazilian cohort, as well as a comparison of the prevalence of pathogenic variants in the general population with that of patients with bronchiectasis, which will provide a better understanding of the magnitude of A1AT deficiency as an etiology of bronchiectasis.

**Chart 1:** Recommendations for alpha-1 antitrypsin testing for patients with bronchiectasis in different guidelines or consensus.

Chalmers et al.^12^	“Alpha-1 antitrypsin testing should not be performed routinely but should be considered in patients with suggestive clinical and radiological features such as basal emphysema or severe airflow obstruction.”
Pereira et al,^15^	Alpha-1 antitrypsin testing is part of the recommended investigation in the algorithm for the diagnosis and etiologic investigation of bronchiectasis.
Hill et al.^13^	“Consider testing for alpha 1 antitrypsin deficiency in patients with coexisting basal panacinar emphysema.”
Chang et al.^14^	“consider the following: Alpha-1-antitrypsin levels if there is evidence of chronic obstructive pulmonary disease/emphysema”
